# DOMINANT BUT NOT NONDOMINANT HANDGRIP ASYMMETRY INFLUENCES ACTIVITY AND COGNITIVE FUNCTION CHANGE

**DOI:** 10.1093/geroni/igae098.3420

**Published:** 2024-12-31

**Authors:** Liangchi Chen, Sae Hwang Han, Jeffrey Lockman

**Affiliations:** The University of Texas Austin, Austin, Texas, United States; University of Texas at Austin, Austin, Texas, United States; The University of Texas Austin, Austin, Texas, United States

## Abstract

Handgrip strength (HGS) is commonly used in clinical and research settings for examining muscle function. Additionally, HGS asymmetry is often used as a screening tool for identifying functional and cognitive limitations among older adults. This longitudinal study examined changes in activity limitations and cognitive function associated with baseline handgrip asymmetry. We analyzed 8 waves of biennial data collected from adults 51 and older in the nationally representative Health and Retirement Study (2006–2020, N=7,053). HGS asymmetry ratio was measured at baseline and was calculated as nondominant HGS/dominant HGS; following earlier works, individuals with HGS asymmetry ratio smaller than 0.80 and higher than 1.20 were considered to have dominant and nondominant handgrip asymmetry, respectively. We used multilevel models to investigate whether HGS asymmetry was linked to a faster rate of change in activity limitations (evaluated with activities and instrumental activities of daily living items; range: 0–11) and cognitive function (evaluated with Telephone Interview for Cognitive Status; range: 0–27) during the observation period. Results indicated that individuals with dominant HGS asymmetry, but not non-dominant HGS asymmetry, developed activity limitations at a faster rate (b=.015, p=.030) and also showed a faster rate of cognitive decline (b=-.053, p=.005) compared to individuals with HGS symmetry. While the present findings are based on a U.S. sample, future research endeavors are aimed at investigating similar issues beyond the confines of the U.S. population.

